# Protocol for a mouse tubuloid model of myoglobinuric acute kidney injury

**DOI:** 10.1016/j.xpro.2025.104205

**Published:** 2025-11-17

**Authors:** Marc Torres Pereiro, Luping Zhou, Thomas Worzfeld

**Affiliations:** 1 Marburg University, Faculty of Medicine, Institute of Pharmacology, Marburg, Germany; 2 Department of Endocrinology and Metabolism, The Affiliated Hospital of Southwest Medical University, Luzhou, China; 3 Metabolic Vascular Diseases Key Laboratory of Sichuan Province, Luzhou, China

**Keywords:** Cell Biology, Molecular Biology, Gene Expression, Organoids, Biotechnology and bioengineering

## Abstract

Myoglobinuric acute kidney injury (AKI) occurs as a result of rhabdomyolysis. Here, we present a protocol based on mouse primary renal tubular epithelial organoids, or “tubuloids”, to model myoglobinuric AKI *in vitro*. We describe how to establish and maintain the culture of mouse tubuloids and how to trigger myoglobin-induced injury. Moreover, we provide instructions on how to quantify tubuloid damage.

For complete details on the use and execution of this protocol, please refer to Zhou et al.^1^

## Highlights

• Step-by-step workflow for establishing and maintaining mouse renal tubuloid cultures
• Instructions on the induction of tubuloid injury by myoglobin application
• Quantification of tubuloid injury by gene expression- and morphology-based readouts

## Abstract (editor highlights)

Publisher’s note: Undertaking any experimental protocol requires adherence to local institutional guidelines for laboratory safety and ethics.

## Graphical abstract

**Figure:**
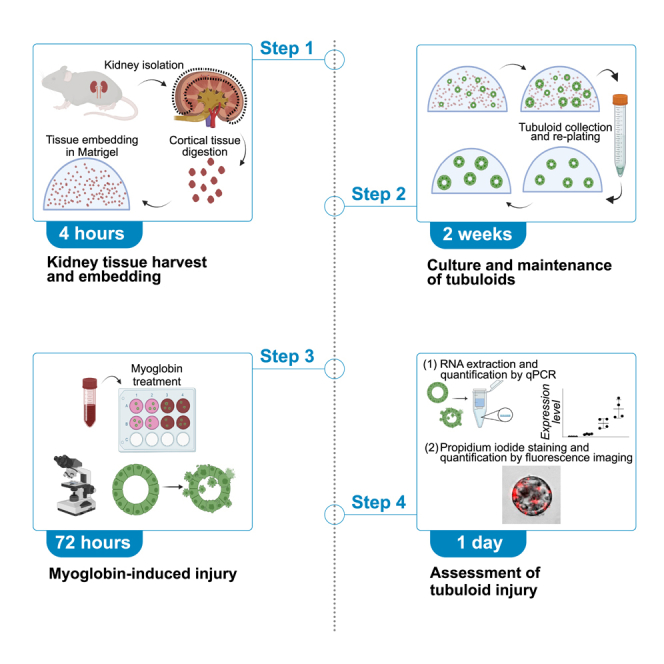

## Before you begin

Organoids are self-organizing three-dimensional structures grown from stem or progenitor cells that recapitulate key features of the tissues they originate from. Importantly, organoids have also become a widely-applied tool to model diseases. For example, primary renal tubular epithelial organoids, termed “tubuloids”, grown from adult kidney tissue have been used to study BK virus infection; moreover, renal “tumoroids” have been derived from Wilms tumors (=nephroblastoma), the most common pediatric solid cancer.^2^ Very recently, renal tubuloids have been employed to model acute kidney injury (AKI), a frequent clinical condition associated with high morbidity and mortality.^3^ AKI can be triggered by various pathological conditions, including rhabdomyolysis, i.e., muscle cell necrosis resulting in release of the muscle protein, myoglobin, which causes acute tubular injury. Tubuloids can be harnessed to identify functionally relevant molecular mechanisms through which renal tubular epithelial cells (mal-)adapt to myoglobin-induced stress.^1^ Moreover, they can be used to assess the effect of pharmacological drugs on myoglobin-induced injury. Here, we describe the experimental steps to establish and propagate renal tubuloid cultures from the mouse renal cortex, and to induce and quantify myoglobinuric injury.

### Innovation

This protocol accurately details, without major modifications, a method that has been previously reported (Zhou et al. 2024^1^). The assessment of tubuloid injury by propidium iodide staining has been optimized, as described here.

### Institutional permissions

Mouse kidney tubuloid experiments were approved by the relevant authorities of the Medical Faculty of Marburg University (AUP AK 21/2021).

Approval from the relevant authorities and animal ethics committees must be obtained prior to performing the protocol described in this article.

### Preparation of myoglobin solution

**Timing: 1 h 45 min**

Myoglobin from equine skeletal muscle is added into the tubuloid culture medium. As only (met)myoglobin (Fe^3+^) is commercially available, myoglobin needs to be reduced to cytotoxic (deoxy)myoglobin (Fe^2+^) before usage in experiments.

1. Weigh myoglobin using a precision scale, and dissolve myoglobin in tubuloid culture medium (materials and equipment) to a final concentration of 20 mg/ml.
  a. Weigh myoglobin powder in a sterile 50 ml tube.
  b. Add tubuloid culture medium. Make sure that no myoglobin powder remains on the wall of the tube, i.e., flush myoglobin from the tube wall.
  c. Incubate at RT for 30 min on a tube roller to dissolve myoglobin. The solution is expected to display a brownish color.

***Note:*** The 50-ml tube has a wide opening, which facilitates the transfer of myoglobin powder from the bottle.

***Alternatives:*** A 15-ml tube can be used when weighing myoglobin amounts lower than 100 mg.

2. Sonicate the solution to further improve myoglobin solubilization. To do so, perform three 5-second sonication cycles (break of 10–15 seconds in between the cycles). During sonication, keep the (tip of the) sonicator inside the solution to prevent excessive bubble formation.

***Note:*** If myoglobin particles are still visible, i.e. if myoglobin is not fully dissolved, incubate the solution at RT for 30 more min on a tube roller.

3. Use L-ascorbic acid (further referred to as Vitamin C) to reduce (met)myoglobin (Fe^3+^) to (deoxy)myoglobin (Fe^2+^).
  a. Add Vitamin C to the solution to a final concentration of 0.75 mM.
  b. Incubate the solution at RT on a tube roller for 1 hour. The solution shall retain a brownish color after the incubation. Troubleshooting 1.

***Note:*** Vitamin C stock solution is prepared by dissolving Vitamin C powder in Ca^2+^- and Mg^2+^-free PBS to a final concentration of 300 mM. Vitamin C stock solution can be aliquoted and stored in the freezer (−20°C) for up to 12 months. To facilitate dissolution of Vitamin C, it is recommended to use warm PBS and incubate 1–2 h on a tube roller.

**CRITICAL:** Vitamin C is light sensitive. Protect Vitamin C solution from light during preparation. Protect the solution (containing myoglobin and Vitamin C) from light during the incubation step.

4. After the incubation step, directly add the final myoglobin solution (containing myoglobin and Vitamin C) to the tubuloids, or store the final myoglobin solution for up to 24 hours in the fridge (4°C) prior to adding it to the tubuloids.

### Preparation of Wnt3a- and R-spondin-1-conditioned media

**Timing: 2–3 weeks**

Wnt3a-producing L cells and R-spondin-1-producing HEK 293T cells are genetically modified to express a zeocin resistance gene. Both cell lines are to be kept in liquid nitrogen for long-term storage.

5. Thaw and grow the Wnt3a-producing and R-spondin-1-producing cells in 10 cm dishes containing DMEM High glucose supplemented with 10% fetal bovine serum (FBS).
6. After 24 h, replace media with zeocin selection media (materials and equipment).
7. Culture both cell lines in zeocin selection media for at least 7 days to select for cells carrying the transgene of interest.

***Note:*** Perform cell splitting if the cells reach 80% confluency during the period of antibiotic selection.

8. After the antibiotic selection, split the cell lines into a 15 cm dish containing DMEM High glucose and 10% FBS.
9. Once reaching 80% confluence, replace the media of the Wnt3a-producing cells and R-spondin-1 producing cells with conditioning media (materials and equipment) and culture them for at least 7 days.

**CRITICAL:** Cells should not be passaged during this period.

10. Collect the conditioned media.
11. In order to remove cells from conditioned media, spin down at 400 g, 5 min at RT and collect the supernatant.
12. Finally filter the conditioned media using a sterile 0.22 μm Filtropur BT 50 filter.

***Note:*** To control for biological activity of Wnt3a- and R-spondin-1-conditioned media, the filtered conditioned media should be analyzed using a TOP-FLASH assay according to published protocols (e.g. https://resources.rndsystems.com/images/site/dw_r-spondinmediumprotocol_34749-web.pdf). In brief, Wnt3a- and R-spondin-1-conditioned media are mixed (30% Wnt3a-conditioned medium, 10% R-spondin-1-conditioned medium, 60% DMEM; i.e. in the same ratio as in tubuloid culture medium, please see below). Induction of TOP-FLASH reporter activity (normalized to Renilla) should be >30 fold.

**Pause point:** Store aliquoted Wnt3a- and R-spondin-1- conditioned media at −80°C for 1 year. After thawing an aliquot of the conditioned media, keep it at 4°C for no longer than 2 weeks.

### Preparation of tubuloid culture media

**Timing: 30 min**

13. Prepare tubuloid culture medium (materials and equipment) under sterile conditions and use within a week after preparation.

### Preparation of kidney digestion buffer

**Timing: 30 min**

Mouse kidney tissue is digested using kidney digestion buffer (materials and equipment). 10 ml of digestion buffer is used to digest a pair of kidneys.

14. Weigh collagenase in a 50 ml tube, and then add DMEM; the final concentration of collagenase is 1 mg/ml.
15. Add deoxyribonuclease (final concentration 60 U/ml) and liberase (final concentration 0.2 mg/ml).

***Note:*** Kidney digestion buffer must be freshly prepared on the day of use.

### Preparation of propidium iodide solution

**Timing: 30 min**

16. Reconstitute propidium iodide powder with DMSO to a concentration of 10 mg/ml.
17. Further dilute with PBS (1:20) to a concentration of 500 μg/ml.
18. On the day of staining, thaw the solution (500 μg/ml) and further dilute with tubuloid culture medium (1:20) to a final concentration of 25 μg/ml.

***Note:*** Propidium iodide solutions should be stored at −20°C and protected from light.

## Key resources table

**Table:** 

REAGENT or RESOURCE	SOURCE	IDENTIFIER
**Chemicals, peptides, and recombinant proteins**		

A83-01	Tocris	2939
Advanced DMEM/F-12	Gibco	12634028
B27 supplement	Gibco	17504044
Ca^2+^- and Mg^2+^-free PBS	Anprotec	AC-BS-0002
Collagenase	Sigma-Aldrich	C4-22
Deoxyribonuclease I from bovine pancreas	Sigma-Aldrich	D5025-15KU
Dimethyl sulfoxide (DMSO)	Sigma-Aldrich	D4540
Dulbecco’s modified Eagle’s medium High glucose	Gibco	C11995500BT
EGF (murine)	Life Technologies	AF-315-09-500μg
Fetal bovine serum Advanced (FBS)	Capricorn	FBS-HI-11A
FGF-10 (rat)	Life Technologies	400-42
Growth factor-reduced Matrigel	Corning	354230
HEPES buffer	Capricorn	HEP-B
L-ascorbic acid 2-phosphate sesquimagnesium salt hydrate (= Vitamin C)	Sigma	A8960
Liberase TL	Roche	540102001
Myoglobin from equine skeletal muscle	Sigma	M0630
N-Acetyl-L-cysteine	Sigma-Aldrich	A9165
Noggin (murine)	PeproTech	250-38
Penicillin/Streptomycin	Gibco	15140122
Primocin	InvivoGen	ant-pm-1
Propidium iodide	Cayman Chemical Company	14289
Stable glutamine 200 mM	Capricorn	STA-B
Zeocin 500 mg	InvivoGen	ant-zn-05

**Critical commercial assays**		

Qiagen RNeasy Mini Kit	Qiagen	74104

**Experimental models: Organisms/strains**		

Mouse (*Mus musculus*): C57BL/6J wild type, male or female, age 9–15 weeks	The Jackson Laboratory	JAX000664

**Experimental models: Primary cells and cell lines**		

Mouse primary renal tubular epithelial cells		
R-spondin-1-producing HEK293T cells	Bio-Techne	3710-001-K
R-spondin-1-producing HEK293T cells (alternative)	Sigma-Aldrich	SCC111
Wnt3a-producing L cells	Hubrecht Institute	NA; based on MTA
Wnt3a-producing L cells (alternative)	ATCC	CRL-2647

**Oligonucleotides**		

*Hmox1* forward (mouse): 5′-CCTGGTGCAAGATACTGCC-3′	Sigma	NA
*Hmox1* reverse (mouse): 5′-GCTCCTTGGTTCTTCCATACAGG-3′	Sigma	NA
*Lcn2* forward (mouse): 5′-CTCAGAACTTGATCCCTGCC-3′	Sigma	NA
*Lcn2* reverse (mouse): 5′-TCCTTGAGGCCCAGAGACTT-3′	Sigma	NA
*Ywhaz* forward (mouse): 5′-TTACTTGGCCGAGGTTGCT-3′	Sigma	NA
*Ywhaz* reverse (mouse): 5′-TGCTGTGACTGGTCCACAAT-3′	Sigma	NA

**Software and algorithms**		

GraphPad Prism 10	GraphPad Software	

**Other**		

Bead Ruptor 4	Biolab products	21-25-010
Centrifuge	Eppendorf	5810R
Ceramic Beads 1.4–1.6 mm Yttrium stabilized	Biolab products	21-25-ZY16.5
Circular vibrating shaker Vibramax 100	Heidolph	544-21200-00
Filtropur BT 50, Bottle top filter, 500 ml, 0.22 μm	Sarstedt	83.3941.511
μ-Slide 8 well	ibidi	80826
Fluorescence microscope Leica Thunder	Leica	NA
MACS smart strainer 70 μm	Miltenyi Biotec	130-098-462
Precision scale	Mettler	AE 200 scale
Sonicator	Bandelin	UW70
Tube roller	neoLab	CAT RM5

## Materials and equipment

### Media composition for Wnt3a-producing L cells

**Table:** Selection medium

Reagent	Final concentration
Dulbecco’s modified Eagle’s medium high glucose	N/A
FBS	10%
Zeocin	125 μg/ml

Store at 4°C for up to 1 month.

**Table:** Conditioning medium

Reagent	Final concentration
Advanced DMEM/F12 medium	N/A
FBS	5%
HEPES buffer	1%
Penicillin/Streptomycin	1%
Stable Glutamine	2 mM

Store at 4°C for up to 1 month.

### Media composition for R-spondin-1-producing HEK293T cells

**Table:** Selection medium

Reagent	Final concentration
Dulbecco’s modified Eagle’s medium high glucose	N/A
FBS	10%
Zeocin	300 μg/ml

Store at 4°C for up to 1 month.

**Table:** Conditioning medium

Reagent	Final concentration
Advanced DMEM/F12 medium	N/A
HEPES buffer	1%
Penicillin/Streptomycin	1%
Stable Glutamine	2 mM

Store at 4°C for up to 1 month.

**Table:** Kidney digestion buffer

Reagent	Final concentration
Dulbecco’s modified Eagle’s medium	N/A
Collagenase	1 mg/ml
Deoxyribonuclease	60 U/ml
Liberase TL	0.2 mg/ml

Keep at 20°C–25°C for up to 12 hours.

***Note:*** 10 ml is used for two kidneys.

**Table:** Tubuloid culture medium

Reagent	Final concentration
Advanced DMEM/F12 medium	N/A
A83-01	5 μM
B27 supplement	1.5%
EGF	50 ng/ml
FGF-10	100 ng/ml
HEPES buffer	10 mM
N-Acetyl-cysteine	1 mM
Noggin	10 ng/ml
Penicillin/Streptomycin	1%
Primocin	0.1 mg/ml
R-spondin-1-conditioned medium	10%
Stable Glutamine	2 mM
Wnt3a-conditioned medium	30%

Store at 4°C for up to 1 week.

**CRITICAL:** N-Acetyl-L-cysteine can cause serious eye and airway irritation. Personal protective equipment needs to be worn according to local health and safety requirements.

***Alternatives:*** Wnt3a- and R-spondin-1-conditioned media can potentially be replaced by recombinant murine Wnt3a and R-spondin-1.

**Table:** Tubuloid freezing medium

Reagent	Final concentration
Advanced DMEM/F12 medium	50%
FBS	40%
DMSO	10%

Store at 4°C for up to 1 week.

(1 ml of freezing medium is used to freeze tubuloids obtained from 2 wells of a 12-well plate.).

## Step-by-step method details

### Establishing mouse renal tubuloid cultures

**Timing:4 h**

This section describes the steps to establish renal tubuloids from mouse kidney cortex tissue.

**Figure 1:**
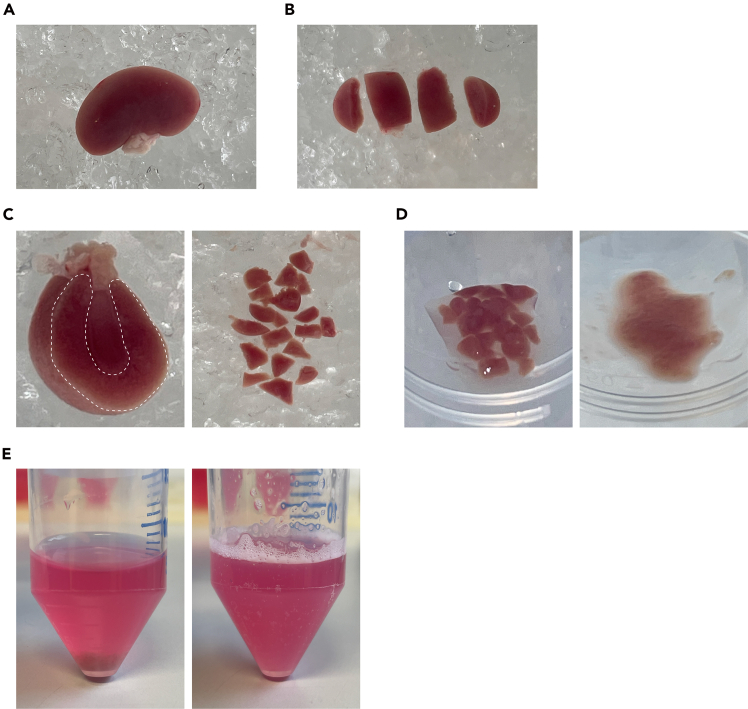
Isolation of kidney cortex (A) Mouse kidney in PBS. (B) A mouse kidney is cut in the transverse plane into 4 segments with a razor blade. (C) The kidney cortex is highlighted in a transverse kidney segment (left; dashed line) and shown after dissection into pieces (right). (D) Kidney cortex pieces in a 50 ml tube before (left) and after mincing with a scissor (right). (E) Minced kidney cortex before (left) and after enzymatic digestion and mechanical fragmentation (right).

***Note:*** Renal tubuloids are derived from kidneys of adult (age 9–15 weeks) male and female C57BL/6J mice.

***Note:*** Rinse kidneys in PBS to remove excess blood. Gently press the kidneys with forceps to remove blood contained inside the kidney.

1. Remove a pair of de-capsulized kidneys^4^ from euthanized mice and keep kidneys in ice-cold PBS (Figure 1A).

2. Cut the kidney into transverse segments using a sharp razor blade (Figure 1B).
3. Dissect the cortex of the kidneys using the razor blade, and cut the cortex into pieces, as small as possible, with the razor blade (Figure 1C).
4. Transfer the cortex pieces to a 50 ml tube. Make sure to transfer the pieces to the wall of the tube, and mince them using sharp scissors (inside the 50 ml tube) (Figure 1D).
5. Wash the minced tissue fragments 2–3 times, until the supernatant is clear of blood.
  a. Add ice-cold PBS until the 15 ml mark.
  b. Centrifuge at 350 g, 4°C for 5 min.
  c. Remove supernatant.
6. Digest the minced kidney cortex fragments with 10 ml of tissue digestion buffer (materials and equipment) for 30 min, at 37°C in a water bath (Figure 1E).

***Optional:*** Shaking of the tube in the water bath during the incubation time improves digestion efficiency.

7. Further reduce the size of the tissue fragments mechanically by vigorous pipetting using a serological 10 ml pipette for 3 min (Figure 1E).

***Note:*** If, after enzymatic digestion and mechanical fragmentation, some macroscopically visible tissue particles are still present, incubate for 5 more min and vigorously pipet again 15 more times.

8. Stop the digestion by adding ice-cold PBS until the 50 ml mark, and filter the cell suspension through a 70 μm cell strainer.
9. Centrifuge at 500 g for 5 min at 4°C.
10. Remove supernatant, add 2 ml of Matrigel.

***Note:*** The obtained cell suspension is sufficient for the culture of 20 wells of a 12-well plate. Reduce the volume of the obtained cell suspension and adjust Matrigel amount accordingly when your experiment requires a lower amount of wells/tubuloids.

***Note:*** Matrigel is stored at −20°C, and it must be thawed and kept on ice all the time to prevent its solidification. A bottle of Matrigel containing 10 ml is recommended to be thawed on ice in the cold room or fridge (4°C). After thawing, it is recommended to be aliquoted (0.5–1 ml), and to be re-frozen for storage.

11. Resuspend cell pellet in Matrigel, and seed five 20 μl droplets per well of a 12-well plate. Troubleshooting 2.
12. Leave the plate under a laminar flow hood for 2–3 min, and avoid moving it. Then, flip the plate upside down to improve Matrigel droplet sphericity and transfer the plate to an incubator (37°C, 5% CO_2_), and let Matrigel solidify for 15 min (Figure 2).

**Figure 2:**
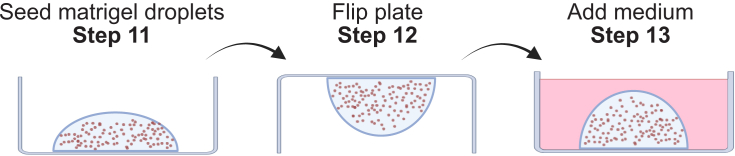
Embedding of cell suspension in matrigel Shown is a schematic illustrating steps 11 to 13 of the protocol.
13. Add 1 ml of pre-warmed tubuloid culture medium per well.
14. Medium needs to be replaced every 2–3 days.

**CRITICAL:** Matrigel liquefies at temperatures below 4°C. Be careful not to add cold solutions to tubuloids in solidified Matrigel.

### Maintaining renal tubuloid cultures

**Timing: 1–3 h**

**Figure 3:**
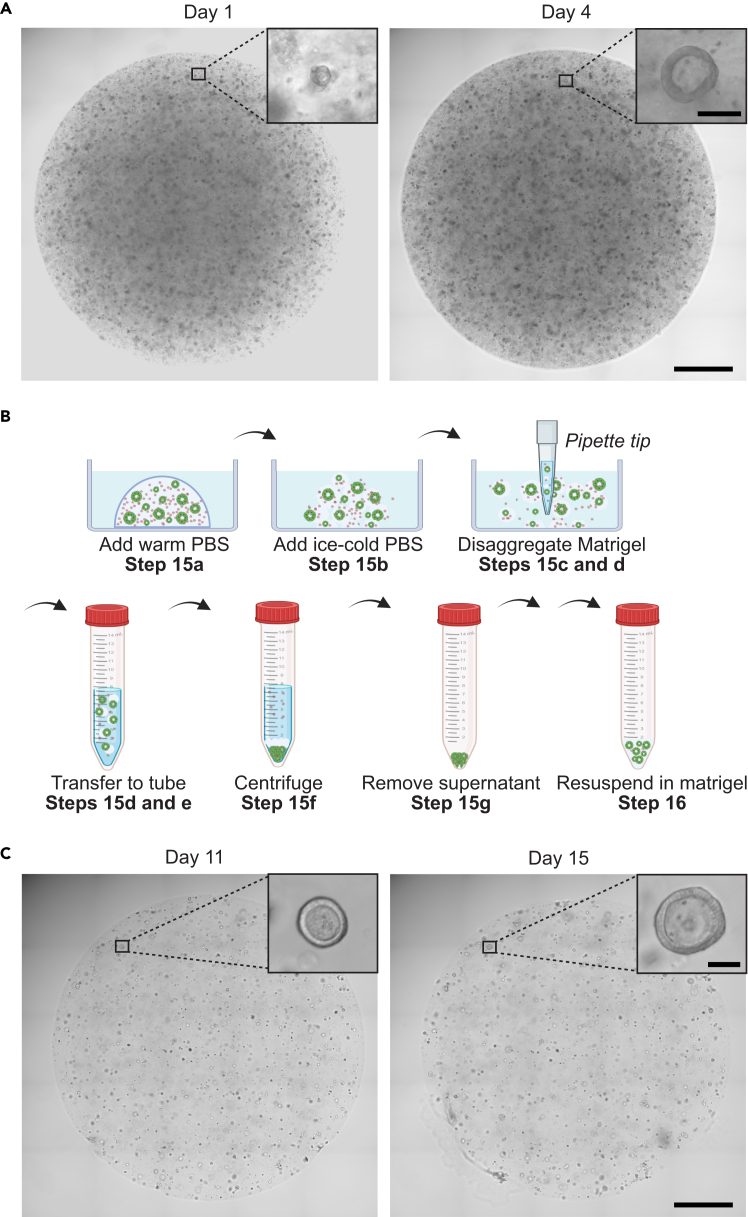
Tubuloid culture (A) Mouse tubuloids 1 day (left) and 4 days (right) after seeding into Matrigel. (B) Shown is a schematic illustrating steps 15 and 16 of the protocol. (C) Mouse tubuloids after one passage, 11 days (left) and 15 days after seeding with Matrigel (right). In A and C, the areas marked with black boxes are magnified in the insets. Shown are bright-field microscopy images. Scale bars: 1 mm, and 50 μm in the insets.

In this section, we describe the steps to maintain and passage renal tubuloid cultures. The first small and immature tubuloid structures can be observed after 1 day in culture (Figure 3A). Mature tubuloids (cuboidal epithelium, defined lumen, polarized structure) can be seen at around day 4 (Figure 3A). Tubuloids should be passaged 6 to 8 days after the initial seeding (Figure 3B).

15. Tubuloid collection.
  a. Remove culture medium and wash the well once with warm PBS (1 ml of PBS per well of a 12-well plate).
    ***Note:*** Use warm PBS to prevent Matrigel fragmentation/liquefaction at this point.
  b. Remove warm PBS and add ice-cold PBS directly onto the Matrigel droplets (1 ml of PBS per well of a 12-well plate).
    ***Note:*** Ice-cold PBS improves cell separation from Matrigel.
  c. Use a 1 ml pipette tip to de-attach the Matrigel droplets from the bottom of the well.
  d. Once de-attached, pipet up and down 10 times to disaggregate the Matrigel droplet into small fragments and transfer the entire content of the well to a 15 ml tube.
  e. Again add 1 ml of ice-cold PBS to the well, and transfer to the same 15 ml tube (in order to recover the entire contents of the well).
    ***Note:*** A maximum of 5 wells of a 12-well plate can be collected in one 15 ml tube.
  f. Add ice-cold PBS until reaching at least the 8 ml mark of the 15 ml tube. Mix the contents of the 15 ml tube by inverting the tube and centrifuge at 350 g at 4°C for 5 min.
    ***Note:*** 1 well of a 12-well plate could be collected into a 1.5-ml Eppendorf tube. However, this is not recommended as low amounts of PBS reduce the efficiency of cell separation from Matrigel.
    ***Note:*** In the 15-ml tube, cells will sink to the bottom, Matrigel will be on top of the cells, and most of the PBS will be on top of the Matrigel.
  g. Remove supernatant containing PBS and part of the Matrigel, add 1 ml of ice-cold PBS and mix 20–25 times to further improve cell separation from the Matrigel.
  h. Add ice-cold PBS until the 8 ml mark and centrifuge (350 g, 4°C, 5 min).
  i. Remove the supernatant. At this point, a cell pellet devoid of Matrigel should be seen.
    **Pause point:** At this step, organoids can be frozen. We recommend freezing the content of two wells of a 12-well plate into one cryovial with 1 ml of tubuloid freezing solution (materials and equipment).
16. Resuspend the cell pellet with Matrigel.

***Note:*** The volume of Matrigel should be twice the volume of Matrigel before passaging, i.e. the “passaging ratio” should be 1:2. For example, if the tubuloids before passaging had been growing in 100 μl of Matrigel, resuspend the cell pellet in 200 μl of Matrigel.

17. Mix well to obtain a homogenous cell suspension (of cells in Matrigel) and seed five 20 μl droplets per well of a 12-well plate. Troubleshooting 2.

***Note:*** Alternatively, three 20 μl droplets can be seeded into one well of a 24-well plate, or one 15 μl droplet can be seeded into one well of a 48-well plate.

18. Leave the plate under a laminar flow hood for 2–3 min, and avoid moving it. Then flip the plate upside down, and transfer the plate to an incubator (37°C, 5% CO_2_), and let Matrigel solidify for 15 min.
19. Add 1 ml of pre-warmed tubuloid culture medium per well.
20. Medium needs to be replaced every 2–3 days.

**Table 1:** Recommended passaging schemes for alternative analyses

Application	Plate format	Passaging ratio	Matrigel drop number and volume	Medium volume
RT-qPCR, RNA-seq, Western Blot	12-well plate	1:2	5 drops, 20 μl each	1 ml
RT-qPCR, RNA-seq	24-well plate	1:2	3 drops, 20 μl each	500 μl
(Immuno-) fluorescence staining	8-well μ-Slide (IBIDI)	1:2 or 1:3	1 drop, 15–20 μl	250 μl
Seahorse assay	96-well plate (Agilent)	2:1 or 1:1	1 drop, 2.5 μl	100 μl

***Note:*** This passaging scheme is optimized for RT-qPCR analyses of tubuloids (as described below). If tubuloids are used in other types of analyses, other passaging schemes may apply (Table 1). For protocols on western blot analyses, immunofluorescence stainings and seahorse assays of tubuloids, please see.^1^^,^^5^^,^^6^

***Note:*** At day 15 of culture (Figure 3C), the number of tubuloids is typically around 200–400 per 20 μl Matrigel drop (if a splitting ratio of 1:2 is used in the first passaging step).

### Myoglobin-induced tubuloid injury

**Timing: 1–3 days**

In this section, we describe how to induce injury of renal tubuloids by myoglobin.

**Figure 4:**
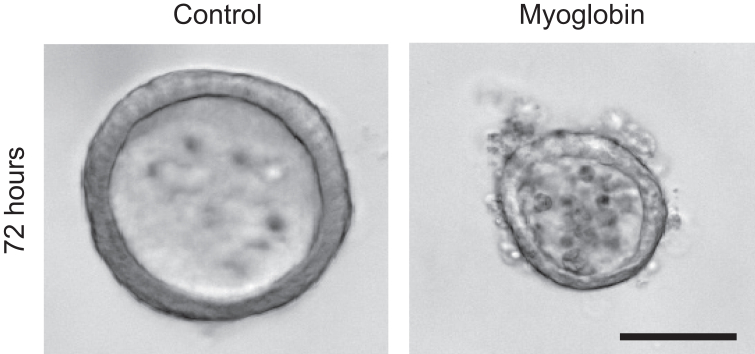
Myoglobin-induced tubuloid injury Bright-field microscopy images of tubuloids treated without or with myoglobin at a concentration of 20 mg/ml for 72 hours. Medium contained 0.75 mM Vitamin C. Scale bar: 50 μm.

***Note:*** Tubuloids can be kept and expanded for several passages. For RT-qPCR and for analyses by fluorescence staining, we recommend to use tubuloids after 1–3 passages (Figure 3C).

***Note:*** For comparison, tubuloids treated with tubuloid culture medium (including Vitamin C, but excluding myoglobin) are used as controls.

***Note:*** Experiments are recommended to include 3–4 biological replicates.

21. 1-4 days after tubuloid passaging, replace tubuloid culture medium with myoglobin solution. After 3 days, tubuloids are expected to exhibit clear morphological signs of damage (Figure 4).

### Assessment of tubuloid injury using RT-qPCR

**Timing: 1 day**

In this section, we describe how to quantify myoglobin-induced injury of renal tubuloids by RT-qPCR.

22. For analysis by RT-qPCR, collect tubuloids as described in Step 15.
23. Resuspend cell pellet in 350 μl of RLT lysis buffer (Qiagen), transfer to 1.5-ml Eppendorf tubes with a safe cap and freeze samples in a −80°C freezer for at least 30 min to increase mRNA yields.

**Pause point:** Samples can be stored in −80°C for several weeks before proceeding with mRNA isolation.

**Figure 5:**
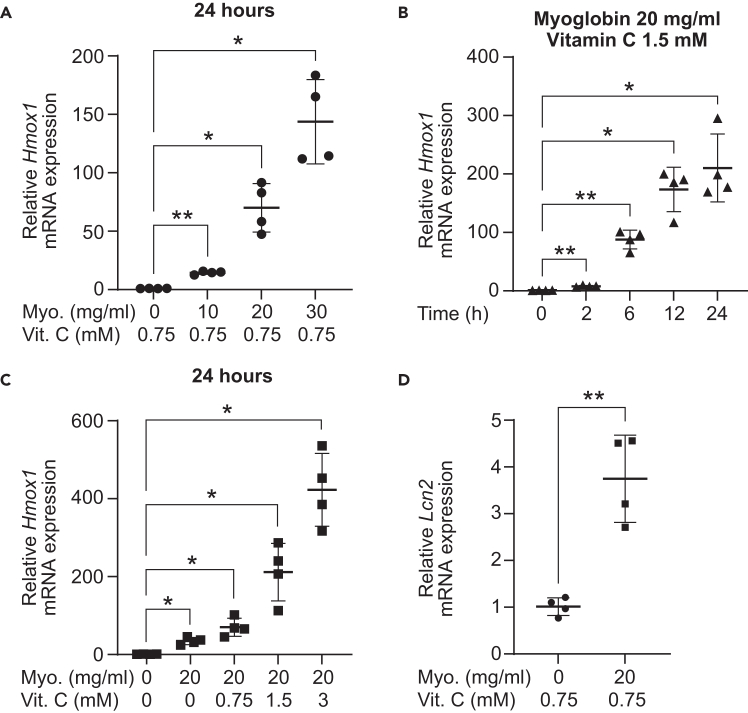
Assessment of myoglobin-induced tubuloid injury by RT-qPCR *Hmox1* and *Lcn2* mRNA expression levels in tubuloids as assessed by RT-qPCR. Tubuloids were treated with (A) 0, 10, 20 and 30 mg/ml of myoglobin, and 0.75 mM of Vitamin C, and analyzed after 24 hours; treated with (B) 20 mg/ml of myoglobin and 1.5 mM of Vitamin C, and analyzed after 0, 2, 6, 12 and 24 hours; treated with (C) 0 or 20 mg/ml of myoglobin, and with 0, 0.75, 1.5 or 3 mM of Vitamin C, and analyzed after 24 hours; treated with (D) 0 or 20 mg/ml of myoglobin, and 0.75 mM of Vitamin C, and analyzed after 24 hours. Data are means ± SD; n = 4 mice; statistical significance has been determined by (A–C) one-way ANOVA, Tukey test or (D) two-sided paired *t* test, and is denoted by ∗*p* ≤ 0.05, ∗∗*p* ≤ 0.01; Myo., myoglobin; Vit. C, Vitamin C.

24. Thaw samples at RT.
25. Add ceramic beads (around 50 beads per sample/1.5-ml Eppendorf tube), transfer 1.5-ml Eppendorf tube to a Bead Ruptor 4.
26. Shake for 1 min at maximum speed for cell lysis.
27. Transfer supernatant (excluding beads) into a fresh 1.5-ml Eppendorf tube.
28. Proceed with mRNA isolation using the Qiagen RNeasy Mini Kit, followed by RT-qPCR for the *Heme oxygenase 1* (*Hmox1*) and *Lipocalin-2* (*Lcn2*) genes (Figure 5).

***Note:****Ywhaz* can be used as a house-keeping gene.^1^

### Assessment of tubuloid injury using propidium iodide staining

**Timing: 1 day**

***Note:*** Assessment of tubuloid injury using propidium iodide staining is independent of the assessment of tubuloid injury using RT-qPCR.

29. Remove cell culture media from the 8-well μ-Slide (IBIDI) and wash the wells with PBS twice (RT, 5 min) in a circular vibrating shaker.
30. Add 200 μl of propidium iodide solution, and incubate the 8-well μ-Slide for 30 minutes in a circular vibrating shaker protected from light.
31. Acquire bright-field and fluorescence images using a microscope connected to a camera (e.g., Leica Thunder).
32. Quantify tubular damage by counting the total number of propidium iodide (PI) foci per tubuloid.

**Figure 6:**
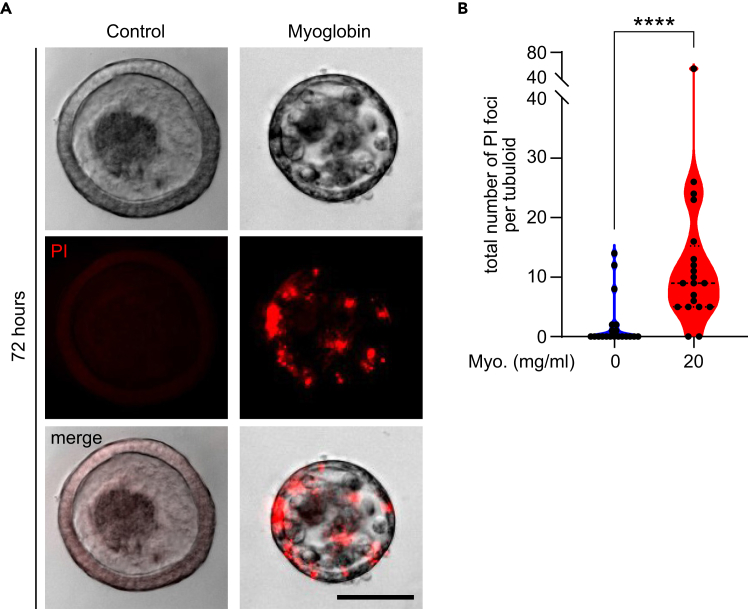
Assessment of myoglobin-induced tubuloid injury by propidium iodide staining Tubuloids were treated without or with 20 mg/ml of myoglobin; medium contained 0.75 mM Vitamin C. After 72 hours, tubuloids were stained with propidium iodide (PI, red). (A) Shown are representative bright-field and fluorescence microscopy images. Scale bar, 50 μm. (B) Quantification of the total number of propidium iodide foci per tubuloid. Data are shown as violin plot with first quartile, median, and third quartile; n=20 tubuloids per condition (derived from one mouse); two-sided unpaired *t* test. Statistical significance is denoted by ∗∗∗∗*p* ≤ 0.0001; Myo., myoglobin; Vit. C, Vitamin C.

***Note:*** A PI focus is defined as discrete, fluorescently labeled point that co-localizes with tubuloid cells (Figure 6).

## Expected outcomes

Tubuloid injury occurs upon exposure to myoglobin in a dose- and time-dependent manner. Morphologically, damaged tubuloids exhibit dead cells and cell debris both in the tubular lumen as well as on the outer surface of the tubuloid (Figure 4). Here, we provide details on how to assess the extent of myoglobin-induced damage using a quantitative method based on *Hmox1* and *Lcn2* gene expression as a readout. Moreover, we describe how to quantify tubuloid injury using a morphological readout based on propidium iodide staining.

Myoglobin is taken up by renal tubular epithelial cells (TECs) and induces TEC injury by increasing the formation of reactive oxygen species and oxidative stress.^7^ Heme oxygenase 1, encoded by the *Hmox1* gene, is a known marker of oxidative stress.^8^ Exposure of tubuloids to myoglobin increases the expression levels of *Hmox1* in a dose- (Figure 5A) and time-dependent manner (Figure 5B).

Vitamin C reduces (met)myoglobin (Fe^3+^) into its cytotoxic form (deoxy)myoglobin (Fe^2+^).^9^^,^^10^ Therefore, the Vitamin C concentration in the medium is an important determinant of myoglobin toxicity: Myoglobin-induced *Hmox1* expression increases with higher concentrations of Vitamin C (Figure 5C). Of note, the effect of Vitamin C on the course of AKI *in vivo* is complex, and anti-oxidant properties of Vitamin C have been linked to beneficial outcomes in different preclinical mouse and rat models of AKI.^11^^,^^12^^,^^13^^,^^14^^,^^15^^,^^16^ On the other hand, excessive Vitamin C has been reported to induce AKI in humans,^17^ potentially in part through indirect mechanisms by causing oxalate nephropathy.^18^

Neutrophil gelatinase-associated lipocalin (Ngal; =Lipocalin-2), encoded by the *Lcn2* gene, is a well-established marker of renal tubular injury,^19^ and can be used for a more direct assessment of myoglobin-induced tubuloid damage (Figure 5D).

Propidium iodide (PI) emits red fluorescence upon binding to double-stranded DNA. Since it cannot pass through plasma membranes of living cells, but can only pass through plasma membranes of injured or dead cells to stain nuclear DNA, it is commonly used to assess cellular damage. Thus, PI staining allows for quantification of tubuloid damage by myoglobin (Figure 6).

## Limitations

The described tubuloid model has some limitations: (1) Tubuloids, as described in this protocol, consist exclusively of epithelial cells. While this facilitates the analysis of epithelial-intrinsic mechanisms of TEC damage and repair, it precludes the study of interactions of epithelial cells with other non-epithelial cell types, such as immune cells. (2) While tubuloids are derived from the renal cortex, which predominantly contains proximal tubular epithelial cells, the exact subtypes of epithelial cells present in tubuloids have not yet been characterized. (3) Tubuloids display an apicobasal polarity, with the apical plasma membrane facing the lumen of the tubuloid, and the basal plasma membrane domain oriented “outwards” (towards the Matrigel). Therefore, the lumen of the tubuloid and the apical plasma membrane of the tubuloid are not readily accessible, e.g., for application of pharmacological drugs from the apical side. (4) In the living kidney *in vivo*, the apical side of TECs is exposed to the flow of the filtrate (produced in the glomerulus). This flow is absent in tubuloids. (5) Under homeostatic conditions in the living organism *in vivo*, TECs of the kidney exhibit very low proliferative activity. In comparison, under the described culture conditions, epithelial cells in tubuloids have higher proliferation rates, thereby not entirely recapitulating a fully homeostatic state, but resembling a (partially) regenerative state.

## Troubleshooting

### Problem 1

**Figure 7:**
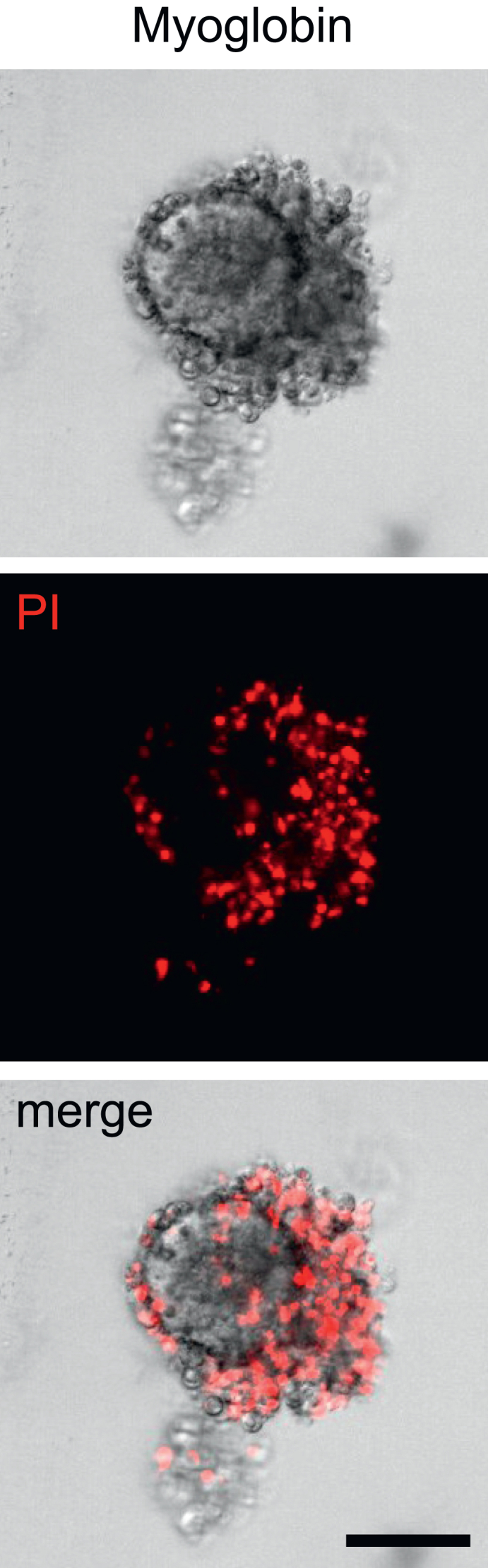
Example of excessive myoglobin toxicity Shown are representative bright-field and fluorescence microscopy images; PI, propidium iodide (red). Scale bar, 50 μm.

The myoglobin solution induces an unexpectedly high cytotoxicity, i.e., morphological signs of excessive cell death can be observed in most of the tubuloids already after 24 hours (Figure 7).

### Potential solution

One possibility for too high cytotoxicity of myoglobin is an excess of Vitamin C. After incubation of myoglobin with Vitamin C (please see “preparation of the myoglobin solution”, Step 3), the solution must remain brownish; if the solution turns red, this is a strong hint for excess of Vitamin C, which results in increased cytotoxicity. Another possibility for too high cytotoxicity is the commercial source of myoglobin, as myoglobin cytotoxicity between different providers can vary. Importantly, we observed also significant batch-to-batch variations. Therefore, it is highly recommended to perform dose-response experiments whenever using a new commercial source or batch of myoglobin; these dose-response experiments should also include different Vitamin C concentrations (as shown in expected outcomes).

### Problem 2

Appearance of bubbles when resuspending renal tissue fragments or tubuloids in Matrigel.

### Potential solution

Matrigel must be thawed and kept on ice before usage. Temperatures higher than 4°C increase the likelihood of bubble formation when resuspending the cell pellet. Additionally, for resuspension and mixing, pipetting should be done slowly and there should always be some liquid remaining both in the tube (1.5 ml or 15 ml tube) and on the pipette tip. If bubbles form, keep the tube on ice until bubbles rise to the surface of the liquid, where they can be removed (using a pipette) or avoided. Finally, during seeding of tubuloids, it is important to keep the tube on ice throughout the procedure to prevent Matrigel solidification and bubble formation.

## Resource availability

### Lead contact

Further information and requests for resources and reagents should be directed to and will be fulfilled by the lead contact, Thomas Worzfeld (worzfeld@uni-marburg.de).

### Technical contact

Technical questions on executing this protocol should be directed to and will be answered by the technical contact, Marc Torres Pereiro (torrespe@staff.uni-marburg.de).

### Materials availability

This study did not generate new unique reagents. Availability of the Wnt3a-producing cell line is under the terms of the Hubrecht Institute.

### Data and code availability

This protocol does not contain datasets or codes.

## Acknowledgments

We thank A. Wüstenhagen, A. Bergen, E. Braun, F. Aktuna, F. Henkel, and M. Losekam for technical assistance and the Hubrecht Institute for kindly providing the cell line for the production of Wnt3a. Figures 2 and 3B as well as the graphical abstract were created with the support of Biorender premium. This work was supported by funds from Marburg University to T.W.

## Author contributions

Conceptualization, T.W.; methodology, M.T.P., L.Z., and T.W.; writing – original draft, M.T.P. and T.W.; writing – review and editing, M.T.P., L.Z., and T.W.; visualization, M.T.P. and T.W.; funding acquisition, T.W.; resources, T.W.; supervision, T.W. All authors have read and agreed to the published version of the manuscript.

## Declaration of interests

The authors declare no competing interests.
